# MicroRNA-138-5p targets the NFIB-Snail1 axis to inhibit colorectal cancer cell migration and chemoresistance

**DOI:** 10.1186/s12935-020-01573-5

**Published:** 2020-10-01

**Authors:** Weifeng Xu, Beibei Chen, Dianshan Ke, Xiaobing Chen

**Affiliations:** 1 Department of Medical Oncology, The Affiliated Cancer Hospital of Zhengzhou University, 127 Dong Ming Road, Zhengzhou, 450008 Henan People’s Republic of China; 2 Department of Cell Biology, Southern Medical University, 510515 Guangzhou, Guangdong China

**Keywords:** Colorectal carcinoma, NFIB-Snail1, Migration, Chemoresistance, miR-138-5p

## Abstract

**Background:**

Colorectal cancer ranks among the most lethal diseases worldwide. Although much progress has been made in research and treatment of colorectal cancer in recent years, the underlying mechanisms related to migration of the cancer cells and the reason for chemoresistance still remain unclear. In this research, we explored the underlying effect of miR-138-5p in colorectal cancer.

**Methods:**

We used qRT-PCR to investigate the expression of miR-138-5p, Snail1, NFIB in colorectal cancer cells. Lentiviral vectors containing miR-138-5p mimics and inhibitors were constructed and transfected cells. Wound healing assay was applied to illustrate interferences on cell migration. Fluorouracial, doxorubicin, cisplat in were used to detect chemotherapy resistance. In order to identify target genes, bioinformatic methods were applied. Snail1 and NFIB protein expression in stable cell lines was detected using Western blot. Double luciferase and CHIP experiment were used to verify binding sites. We used rescue experiments to further explore the interactions among Snail1, NFIB and miR-138-5p.

**Results:**

The expression of miR-138-5p in colorectal cancer cells was low. miR-138-5p inhibited cell migration in colorectal cancer, and could negatively regulate chemotherapy resistance. miR-138-5p targeted NFIB, and regulated Snail1 expression, which mediated colorectal cancer cell migration and chemotherapy resistance.

**Conclusions:**

Our research indicates that miR-138-5p could be a crucial modulator controlling colorectal cancer cell migration and chemoresistance, by acting upon the NFIB-Snail1 axis. miR-138-5p has an emerging prospect to be exploited as a new target for colorectal cancer.

## Background

Cancer is in the forefront of threatening the public health globally [1]. Colorectal carcinoma ranks the fourth among the most lethal types. Smoking, alcohol, lack of exercise, and overweight are contributing factors to the genesis and poor prognosis of colorectal cancer. Its morbidity and mortality can be mitigated through proper screening and surveillance [2]. Treatments for colorectal cancer include endoscopic and surgical procedures, chemotherapy and radiotherapy [3]. Colonic stenting is also a palliative therapy for unresectable colon cancer [4]. In order to find better treatment options, it is increasingly important to discover novel molecular targets and mechanisms for colorectal cancer.

miRNAs could modulate several important biological processes, such as regulating the expression of cancer initiative and repressive genes. In recent years, miR-138-5p was found to reduce cancer growth in several cancers [5–7]. For example, research suggested that in pancreatic cancer, miR-138-5p had a suppressing role [8]. Gao et al’s research showed that through targeting EIF4EBP1, it had an impact on nasopharyngeal cancer radiotherapy [9]. Roberto et al. found that the miR-138-5p expression was related to osteosarcoma prognosis [10]. Zhao et al. found that by targeting RHBDD1, it could mitigate progression of breast cancer [11]. Zhu et al’s research showed that it intervened in the development of lung cancer [12].

In this study, we explored the underlying mechanisms for the roles of miR-138-5p in colorectal cancer migration and chemoresistance. We found that expression of miR-138-5p was lower in colorectal cancer. We also found that miR-138-5p inhibited colorectal cancer cell migration and chemoresistance. NFIB was discovered as a target gene by using bioinformatics methods. NFIB and Snail1 were positively correlated. We subsequently performed knock down experiments to define the interactions among miR-138-5p, NFIB and Snail1. Our research provided new insights towards the treatment of colorectal cancer.

## Materials and methods

### Patients and samples

This study was approved by the ethics committee of the Affiliated Cancer Hospital of Zhengzhou University. 100 patients diagnosed with colorectal cancer were selected. All patients had read and signed the informed consent. Cancerous tissues and adjacent corresponding normal tissues were obtained. Samples were immediately frozen and stored in the refrigerator at − 80 ℃. Clinical and pathological features of 100 patients were presented in the Table 1.

**Table 1:** Clinical and pathological features of patients

Features	Number of patients
Age	
≦ 50	41
> 50	59
Sex	
Male	62
Female	38
Lymph node status	
N0	55
N1	39
N2	6
Tumor stage	
I–II	79
III–IV	21

### Bioinformatic methods

We used miRDB (http://mirdb.org/) [13], DIANA (http://diana.imis.athena-innovation.gr/) [14], TargetScan (www.targetscan.org) [15] for predicting target genes. GEPIA (http://gepia.cancer-pku.cn/) [16] was utilized to verify correlation between NFIB mRNA and Snail1 mRNA.

### Cell culture

We bought five human colorectal cancer cell lines (LOVO, HCT116, HT29, SW620, SW480) and one normal cell line (FHC) from ATCC (Virginia, USA). All cells were grown in RPMI 1640 (Hy-clone, USA) medium, antibiotics and 100 U/ml penicillin and 100 μg/ml streptomycin 10% fetal serum (FBS, Gipco, USA) were added. The cells were cultured in an incubator at a temperature of 37 °C with 5% CO_2_ in a humid atmosphere.

### qRT-PCR

Total RNA was isolated using TRIzol reagent (Invitrogen Life Technologies, USA) and then was reverse-transcribed into cDNA with the Primer Script RT reagent kit (Takara Bio, China). We used SYBR Green PCR Kit (TAKARA, Japan) to perform qRT-PCR. We used U6 and GAPDH as internal controls. The forward primer for miR-138-5p was: 5′-CTAGAGCTCAACTGAAGTGGCTAAACTG -3′ and reverse primer was 5′-GCTAGGCGTTGAAGTTCTGCCTAAATGC-3′. The forward primer for NFIB was: 5′-GCTGTGTCTTATCCAATCCCG-3′ and reverse primer was 5′-TGCCTTTGAACAGGATCACCA-3′. The forward primer for Snail1 was 5′-TCGGAAGCCTAACTACAGCGA-3′, and reverse primer was 5′-AGATGAGCATTGGCAGCGAG-3′. The mRNA levels were analyzed using 2^−ΔΔCt^ method. Three times were replicated for each sample with no RT and no template control.

### Western blot

We used 1% PMSF and RIPA lysine buffer to confine cellular protein, then reacted with the SDS-PAGE test buffer. Proteins were transferred to a polyvinylide difluoride layer (Millipur, USA). The layer was grown overnight, after incubation for 1 h, at room temperature. Then we applied the ECL chemiluminescence kit (Advansta, USA), and proteins were fried with secondary antibodies for 1 h. We examined bands using GeneGnome 5 (Synoptics Ltd., UK).

### Wound healing cell migration assay

We used Oris Cell Migration Kit (Platypus, USA) to assess cell migration. Cells in the 6-well plates grew into full confluence. By scratching the monolayer with a 200-µl plastic pipette tip, a tiny area was disrupted. We washed the cells twice using phosphate-buffer saline (PBS), then replaced using a complete medium, which contained various concentrations of API. We observed wound closure after 48 h. Under a phase-contrast microscope at 100× magnification, pictures were captured immediately. Cells were washed with PBS.

### CCK-8 assay

We used cell count-Kit-8 (CCK-8, Dojinto, Japan). We digested the stable strain with 1 ml of 0.25% trypsin. Then it was transferred to a 15 ml centrifugal tube, later it was centrifuged at 1500 rpm for 5 min. We seeded the cells in six-well plates. After 48 h, we detected cell viability using CCK8. In short, 10 µl of 5 mg/ml CCK8 solution was added to the 96‐well plate. Then it was incubated for 2 h in the dark. In the microplate, the absorbance of each well was measured at 450 nm.

### Generation of lentiviruses and stable transfection

miR-138-5p mimic and its parallel negative control (mNC), miR-138-5b inhibitor and parallel negative control (inhibitor NC) were purchased from RiboBio (Guangzhou, China). Vectors were transfected, plasmids packaged into cells using Polyget Regent (Signage Labs, USA). After 48 h, virus supernatants were collected. In 12-well plates, cancer cells were spin-infected. Then we centrifuged lentiviral supernatants for half an hour. In order to harvest stable cell lines, cells were selected using puromycin (3 ng/ml). Transient transfections were conducted using Lipofectamine 2000 (Invitrogen, Karlspot, CA, USA).

### ChIP-seq analysis and dual-luciferase reporter assay

We cross-linked cells using the Qiagen PCR Purification kit. We scraped cell monolayers in soft lysis buffer. Then we lysed nuclei pelletsusing SDS lysine buffer. Protein was diluted 1:10 with dilution buffer and precleared by incubating with protein magnetic beads (Millipore). We immunoprecipitated the samples using the initial antibody. Antibody-chromatin complexes were rescued. Then we washed the beads with buffers. The eluted samples were decrosslinked. Next day, we treated the proteins complexes with proteinase K solution, then incubated them for an hour. We used dual luciferase reporter gene assay kit (Yeasen, China) according to the instruments provided by manufacture.

### Statistical analysis

Data was presented as mean ± S.D. We used GraphPad Prism 8.0 software and the SPSS25.0 software to conduct all the statistical analyses. Significant difference between each group was analyzed using unpaired 2-tailed Student’s t tests or one-way ANOVA. A *p* value < 0.05 was considered statistically significant.

## Results

### Expression of miR-138-5p was reduced in colorectal cancer tissues

**Fig. 1:**
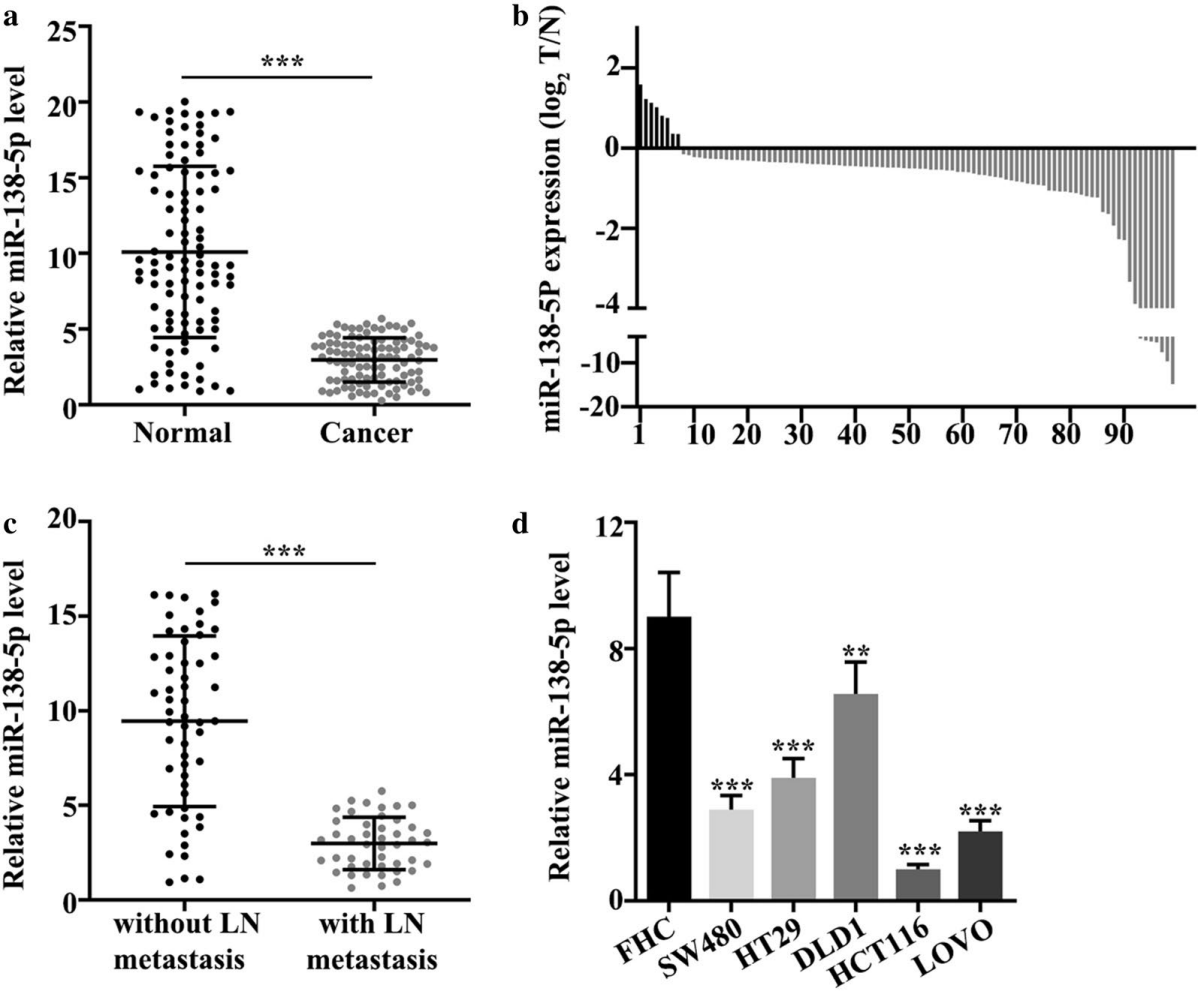
Expression of miR-138-5p was reduced in colorectal cancer tissues. **a** miR-138-5p expression was low in colorectal cancer (p < 0.001). **b** Among 100 samples, 8 were high and 92 were low in expression of miR-138-5p. **c** Expression in tissues with lymph node metastasis was low, while expression in samples without lymph node metastasis was high (p < 0.001). **d** Normal colonic mucosal epithelial cells were higher in expression than five colorectal cancer cell lines (p < 0.01)

In 100 colorectal cancer samples, expression of miR-138-5p was significantly reduced (p < 0.001) (Fig. 1a). Figure 1b illustrated that 8 tumor tissues were high and 92 were low in expression. Then, we analyzed expression of miR-138-5p in colorectal cancer samples with and without lymph node metastasis. Results revealed that expression of miR-138-5p in tissues with lymph node metastasis was low, while expression in samples without lymph node metastasis was high (p < 0.001) (Fig. 1c). We further detected miR-138-5p expression in normal colonic cells and 5 colorectal cancer cell lines: FW480, HT29, DLD1, HCT116, LOVO. Results revealed that the expression was decreased in the colorectal cancer cell lines (p < 0.01) (Fig. 1d).

### miR-138-5p inhibited cell migration in colorectal cancer

**Fig. 2:**
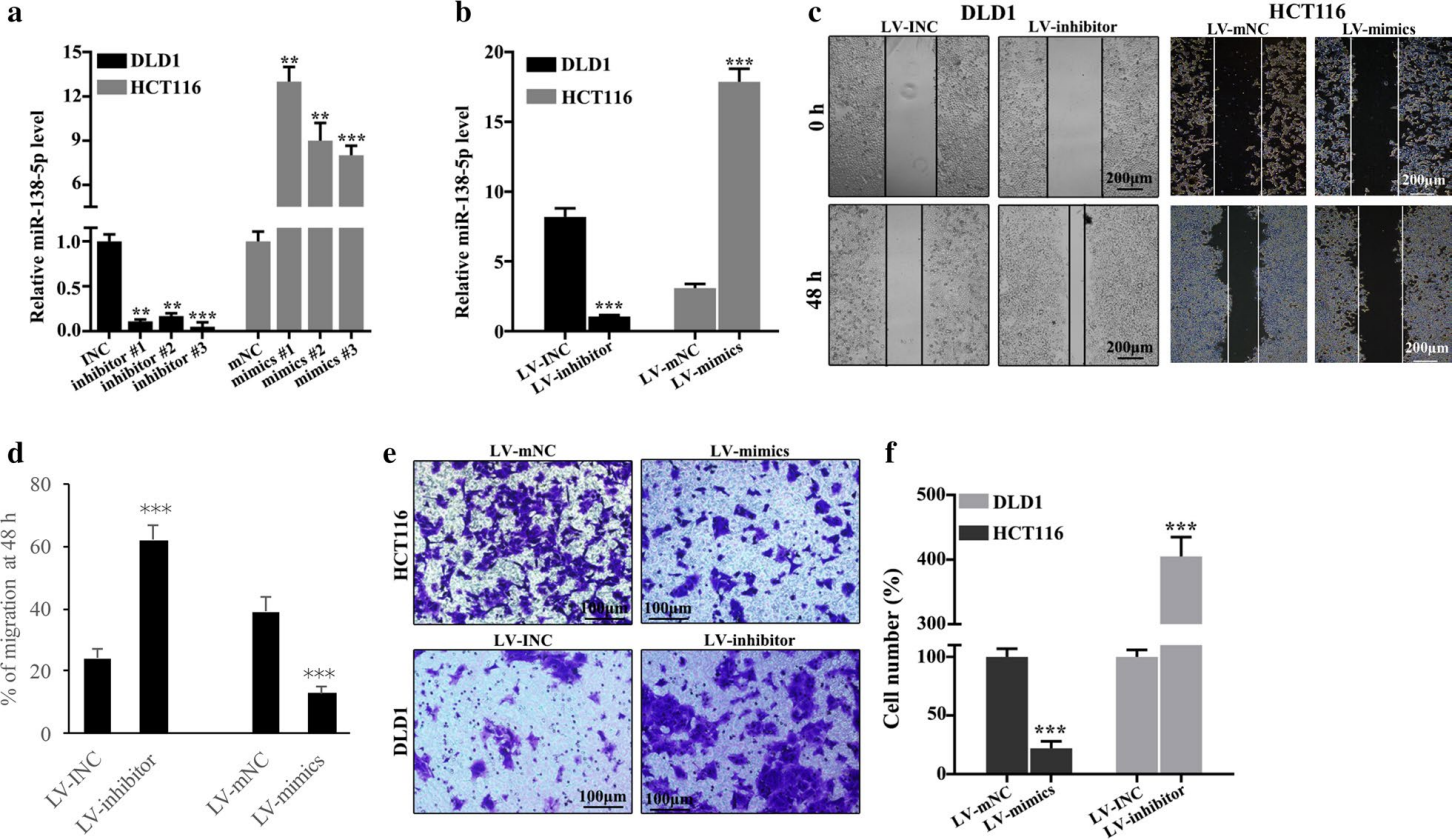
Cell migration in colorectal cancer was inhibited by miR-138-5p. **a** The best mimic and inhibitor sequences were screened out. **b** Effectiveness of lentiviral transfection in DLD1 and HCT116 cell lines were verified. p < 0.001. **c** Cell migration was detected by scratch experiment. **d** In DLD1 cell lines, LV-inhibitor group exhibited higher percentage of cell migration than LV-INC group (p < 0.001). **e**, **f** In HCT116 cell lines, the LV-mimics group was lower in the percentage of cell number compared with LV-mNC group (p < 0.001). In DLD1 cell lines, the LV-INC group was lower in the percentage of cell number compared with LV-inhibitors group (p < 0.001)

For further probing the functionality of miR-138-5p in colorectal cancer cells, we knocked down its expressions in DLD1 cells and enhanced its expression in HCT116 cells. Successful knockdown of miR-138-5p could be observed in DLD1 and successful up-regulation in HCT116 cells (p < 0.001). We screened out the best mimic and inhibitor sequences (Fig. 2a). To verify the effectiveness of lentivirus transfection, we performed PCR. Compared with control groups, lentiviral vectors containing miR-138-5p inhibitors (LV-inhibitor) group was lower, while the lentiviral vectors containing miR-138-5p mimics (LV-mimic) group was higher in expression of miR-138-5p (p < 0.001) (Fig. 2b). Knockdown treated DLD1 cells were examined by scratch test at 0 h and 48 h. Figure 2c illustrated that depleted expression of miR-138-5p significantly increased cell migration at 48 h. Cell migration analysis suggested knockdown treatment could increase the DLD1 cell migration (p < 0.001) (Fig. 2d). In HCT116 cell lines, group of LV-mimics was lower in the percentage of cell number compared with LV-mNC group (p < 0.001). In DLD1 cell lines, group of LV-inhibitors increased the percentage of cell number compared with LV-INC group (p < 0.001) (Fig. 2e, f).

### miR-138-5p inhibited chemotherapy resistance of colorectal cancer cells

**Fig. 3:**
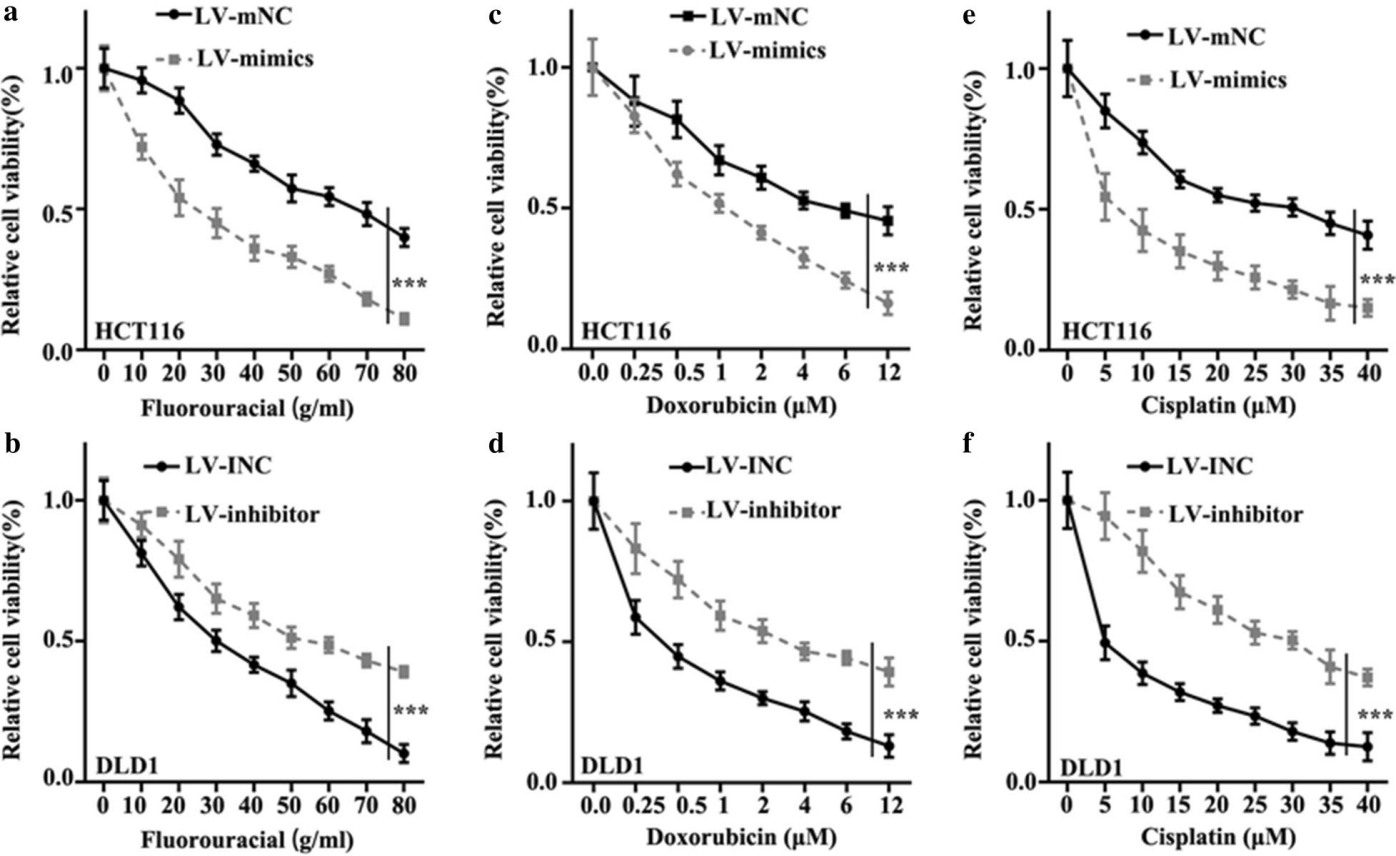
miR-138-5p could inhibit chemotherapy resistance of colorectal cancer cells. **a** In Fluorouracial treated HCT116 cell lines, the relative cell viability of LV-mimics group was lower than LV-mNC group (p < 0.001). **b** In Fluorouracial treated DLD1 cell lines, LV-inhibitor group was higher than LV-INC group in cell viability (p < 0.001). **c** In Doxorubicin treated HCT116 cell lines, LV-mimics group was lower than LV-mNC group (p < 0.001). **d** In Doxorubicin treated DLD1 cell lines, LV-inhibitor group was higher than LV-INC group (p < 0.001). **e** In Cisplatin treated HCT116 cell lines, LV-mimics group was lower than LV-mNC group (p < 0.001). **f** In Cisplatin treated DLD1 cell lines, LV-inhibitor group was higher than LV-INC group (p < 0.001)

Three chemotherapy drugs (Fluorouracial, doxorubicin, cisplatin) were used to detect chemotherapy resistance of colorectal cancer cells. For Fluorouracial, in HCT116 cells lines, LV-mimics group was lower in relative cell viability than LV-mNC group (p < 0.001) (Fig. 3a), in DLD1 cell lines, LV-inhibitor group was higher in relative cell viability than LV-INC group (p < 0.001) (Fig. 3b). For Doxorubicin, in HCT116 cells lines, LV-mimics group was lower in relative cell viability than LV-mNC group (p < 0.001) (Fig. 3c), in DLD1 cell lines, LV-inhibitor group was higher in relative cell viability than LV-INC group (p < 0.001) (Fig. 3d). For Cisplatin, in HCT116 cells lines, LV-mimics group was lower in relative cell viability than LV-mNC group (p < 0.001) (Fig. 3e), in DLD1 cell lines, LV-inhibitor group was higher in relative cell viability than LV-INC group (p < 0.001) (Fig. 3f).

### miR-138-5p modulated Snail1 expression, which mediated colorectal cancer cell migration and chemotherapy resistance

**Fig. 4:**
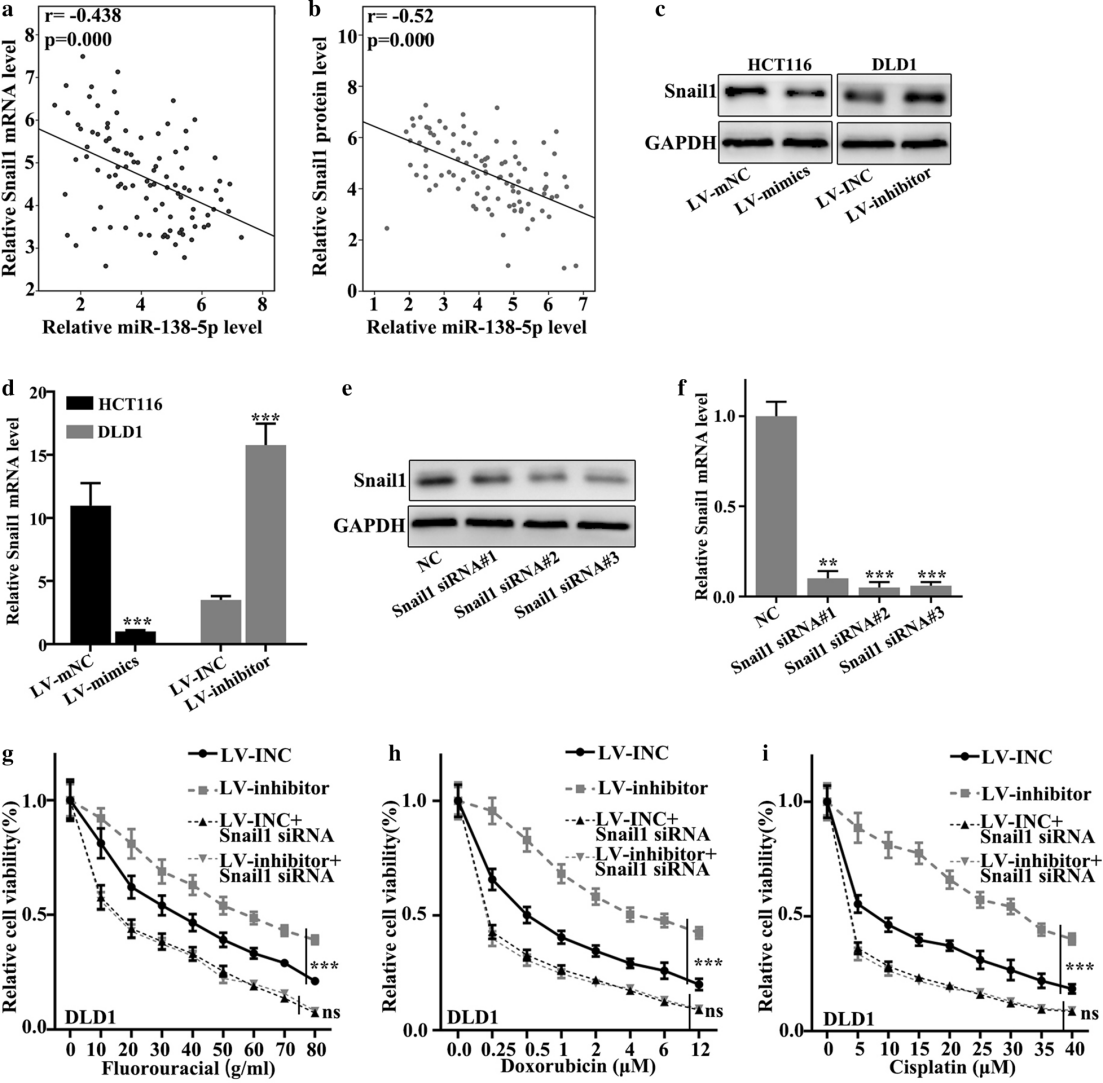
Snail1 expression mediated colorectal cancer cell migration and chemotherapy resistance and was regulated by miR-138-5p. **a** Relative Snail1 mRNA level negatively correlated with relative miR-138-5p level (r = − 0.438, p < 0.001). **b** Relative Snail1 protein level negatively correlated with relative miR-138-5p level (r = − 0.52, p < 0.001). **c** Snail1 protein expression in stable cell lines. **d** In HCT116 cell lines, LV-mimics group was lower in the expression of Snail1 mRNA than LV-mNC group (p < 0.001). In DLD1 cell lines, LV-inhibitors group was higher in the expression of Snail1 mRNA than LV-INC group (p < 0.001). **e**, **f** Snail1 siRNA sequences with the best interference effect was screened for subsequent experiments. **g** In Fluorouracial treated DLD1 cell lines, the difference of relative cell viability between LV-inhibitor + Snail1 siRNA group and LV-INC + Snail1 siRNA group showed no statistical significance (p > 0.05). **h** In Doxorubicin treated DLD1 cell lines, the difference of relative cell viability between LV-inhibitor + Snail1 siRNA group and LV-INC + Snail1 siRNA group was not statistically significant (p > 0.05). **i** In Cisplatin treated DLD1 cell lines, the difference of relative cell viability between LV-inhibitor + Snail1 siRNA group and LV-INC + Snail1 siRNA group was not statistically significant (p > 0.05)

We detected the relationship between miR-138-5p and Snail1 mRNA and Snail1 protein expression in 100 clinical samples. Figure 4a showed that relative Snail1 mRNA level negatively correlated with level of miR-138-5p (p < 0.001) Fig. 4b showed that relative Snail1 protein level negatively correlated with level of miR-138-5p (p < 0.001). Then we examined Snail1 protein expression in stable cell lines (Fig. 4c). In HCT116 cell lines, LV-mimics group was lower in the expression of Snail1 mRNA than LV-mNC group (p < 0.001). In DLD1 cell lines, LV-inhibitors group was higher in the expression of Snail1 mRNA than LV-INC group (p < 0.001) (Fig. 4d). Snail1 siRNA sequences with the best interference effect was screened for subsequent experiments (Fig. 4e, f). In Fluorouracial treated DLD1 cell lines, the difference of relative cell viability between LV-inhibitor + Snail1 siRNA group and LV-INC + Snail1 siRNA group showed no statistical significance (p > 0.05) (Fig. 4g). In Doxorubicin treated DLD1 cell lines, the difference of relative cell viability between LV-inhibitor + Snail1 siRNA group and LV-INC + Snail1 siRNA group displayed no significance statistically (p > 0.05) (Fig. 4h) In Cisplatin treated DLD1 cell lines, the difference of relative cell viability between LV-inhibitor + Snail1 siRNA group and LV-INC + Snail1 siRNA group was not statistically significant (p > 0.05) (Fig. 4i). No significant difference was observed between group LV-inhibitor + Snail1 siRNA and group LV-INC + Snail1 siRNA in terms of cell viability (Fig. 4g–i).

### miR-138-5p targeted NFIB

**Fig. 5:**
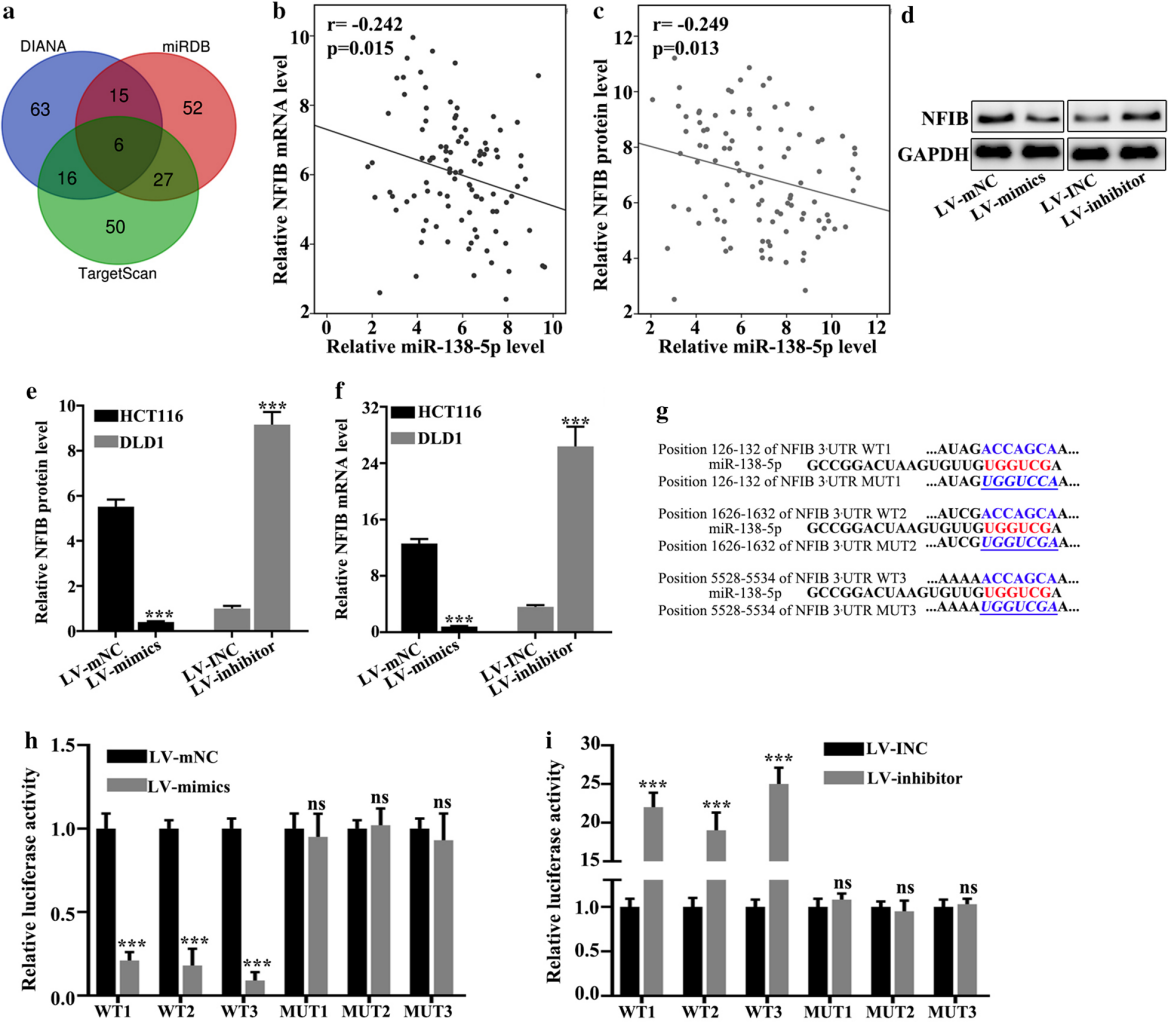
miR-138-5p targeted NFIB. **a** Target genes of miR-138-5p were predicted by three databases, and NFIB was screened out based on biological function. **b** Relative NFIB mRNA level negatively correlated with relative miR-138-5p level (r = − 0.242, p < 0.05). **c** Relative NFIB protein level negatively correlated with relative miR-138-5p level (r = − 0.249, p < 0.05). **d** NFIB protein expression in stable cell lines. **e** In HCT116 cell lines, LV-mimics group was lower in the expression of NFIB protein than LV-mNC group (p < 0.001). In DLD1 cell lines, LV-inhibitors group was higher in the expression of NFIB protein than LV-INC group (p < 0.001). **f** In HCT116 cell lines, LV-mimics group was lower in the expression of NFIB mRNA than LV-mNC group (p < 0.001). In DLD1 cell lines, LV-inhibitors group was higher in the expression of NFIB mRNA than LV-INC group (p < 0.001). **g** The wild-type and mutant plasmids of NFIB mRNA 3 UTR were constructed on the potential binding sites of miR-138-5p and NFIB mRNA. **h** Double luciferase assay verified that miR-138-5p banded with wild type NFIB mRNA 3 UTR instead of mutant type (p < 0.001). **i** The NFIB siRNA with the best interference effect sequences was screened for subsequent experiments

Target genes were explored using three databases (DIANA, TargetScan, miRDB). NFIB was screened out based on biological function (Fig. 5a). Figure 5b illustrated that relative NFIB mRNA level negatively correlated with relative miR-138-5p level (p < 0.05). Figure 5c illustrated that relative NFIB protein level negatively correlated with relative miR-138-5p level (p < 0.05) Then we examined NFIB protein expression in stable cell lines (Fig. 5d). In HCT116 cell lines, LV-mimics group was lower in the expression of NFIB protein than LV-mNC group (p < 0.001). In DLD1 cell lines, LV-inhibitors group was higher in the expression of NFIB protein than LV-INC group (p < 0.001) (Fig. 5e). In HCT116 cell lines, LV-mimics group was lower in the expression of NFIB mRNA than LV-mNC group (p < 0.001). In DLD1 cell lines, LV-inhibitors group was higher in the expression of NFIB mRNA than LV-INC group (p < 0.001) (Fig. 5f). The wild-type and mutant plasmids of NFIB mRNA 3 UTR were constructed on the potential binding sites of miR-138-5p and NFIB mRNA (Fig. 5g). Double luciferase assay verified that miR-138-5p banded with wild type NFIB mRNA 3 UTR instead of mutant type (p < 0.001) (Fig. 5h). The NFIB siRNA with the best interference effect sequences was screened for subsequent experiments (Fig. 5i).

### NFIB activated Snail1

**Fig. 6:**
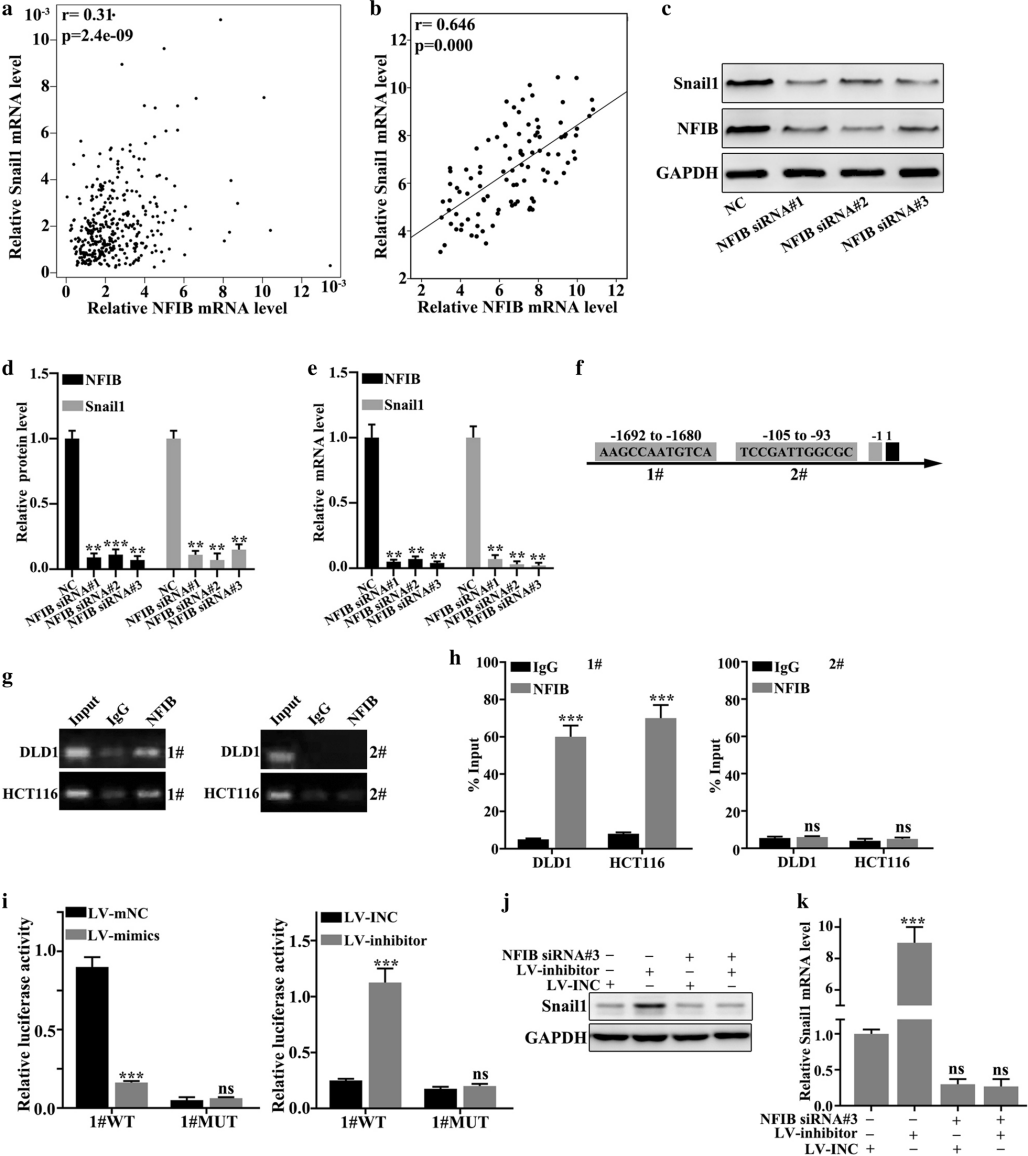
NFIB activated Snail1. **a** NFIB mRNA and Snail1 mRNA expression had positive correlation in 100 colorectal cancer tissues (r = 0.31, p < 0.001). **b** In GEPIA database, NFIB mRNA and Snail1 mRNA expression were positively correlated (r = 0.646, p < 0.001). **c** Expression of Snail1 and NFIB after transfected with NFIB siRNA. **d** After transfected with NFIB siRNA, the relative protein level of Snail1 were decreased (p < 0.01). **e** After transfected with NFIB siRNA, the relative mRNA level of Snail1 were decreased (p < 0.01). **f** CHIP experiment verified that transcription factor NFIB could bind to the promoter region of Snail1 gene. **g**–**i**. According to the binding sites predicted by the database, wild-type and mutant plasmids of the Snail1 promoter region were constructed. Double luciferase assay experiment verified that NFIB binded to the wild-type promoter region of Snail1, instead of the mutant type. **j**–**k** Expression of Snail1 was detected after co-transfected NFIB siRNA into stable cell lines. The difference was statistically significant between NFIB siRNA-LV inhibitor + LV INC− group and NFIB siRNA-LV inhibitor-LV INC + group (p < 0.001) The difference was not statistically significant between NFIB siRNA + LV inhibitor-LV INC + group and NFIB siRNA + LV inhibitor + LV INC− group (p > 0.05)

NFIB mRNA and Snail1 mRNA expression were in positive correlation in 100 colorectal cancer tissues (p < 0.001) (Fig. 6a). In GEPIA database, NFIB mRNA and Snail1 mRNA expression were positively correlated (r = 0.646, p < 0.001) (Fig. 6b). Expression of Snail1 and NFIB after transfected with NFIB siRNA (Fig. 6c). After transfected with NFIB siRNA, the relative protein level of Snail1 were decreased (p < 0.01) (Fig. 6d). After transfected with NFIB siRNA, the relative mRNA level of Snail1 were decreased (p < 0.01) (Fig. 6e). CHIP experiment verified that transcription factor NFIB could bind to the promoter region of Snail1 gene (Fig. 6f). According to the binding sites predicted by the database, wild-type and mutant plasmids of the Snail1 promoter region were constructed. Double luciferase assay experiment verified that NFIB banded to the wild-type promoter region of Snail1, instead of the mutant type (Fig. 6g–i). Expression of Snail1 was detected after co-transfected NFIB siRNA into stable cell lines. The difference was statistically significant between NFIB siRNA-LV inhibitor + LV INC− group and NFIB siRNA-LV inhibitor-LV INC+ group (p < 0.001) The difference was not statistically significant between NFIB siRNA + LV inhibitor-LV INC+ group and NFIB siRNA + LV inhibitor + LV INC− group (p > 0.05) (Fig. 6j–k).

## Discussion

A large proportion of cancer mortality is attributed to colorectal cancer, and the incidence of colorectal cancer in younger adults has been on the rise [17, 18]. Although much progress has been made to invent new treatments for combating colorectal cancer, molecular mechanisms of factors that contribute to poor prognosis such as metastasis and drug resistance are still not elucidated. Therefore, finding the key effect or that modulate colorectal cancer metastasis and drug resistance is crucial. It was notable that miR-138-5 manifested important roles in regulating tumorigenesis and progression in several types of cancers [19]. As is highlighted in the literature that miR-138-5p could function as an effective tumor suppressor and monitor cell cycle. Meanwhile, it also participates in other biological processes such as neuronal development [20], autophagy and so on [21].

NFIB is a member of the nuclear factor family (which also includes NFIA, and NFIX). Emerging evidence has demonstrated that NFIB could not only participate in normal somatic development [22], it also acted as an oncogene and involved in tumorigenesis of several cancers, including osteosarcoma [23], glioma [24], astrocytoma [25], gastric cancer [26], breast cancer [27]. Liu et al’s research showed that NFIB could suppress p21 transcription [28]. NFIB could also mediate the accessibility of chromatin [29]. The SNAIL1 gene is located in chromosome 20q.13.13. It contains three exons encoding 264 amino acids [30]. Through repressing the adherent and tight junctions, Snail1 could effectively promote the epithelial–mesenchymal transition in epithelial cells, leading to cell migratory and tumor progression [31]. Emerging evidence has suggested that Snail1 acted as an important regulator of epithelial-mesenchymal transition. Snail1 could inhibit molecules such as E-cadherin and claudins (cell adhesion related), and promote cell migratory capacity. Cell adhesion, polarity and migratory properties change during epithelial–mesenchymal transition. SNAIL1 also participates in several crucial signaling pathways, such as RTKs, TGFβ, NOTCH, WNT, TNF α and BMPs pathways [32]. Study by Frey et al. demonstrated that Snail1 employed canonical BMP signaling in colorectal cancer [33]. Literature demonstrates that Snail1 is a key effector in modulating biological process in breast cancer [34, 35], pancreatic cancer [36], lung cancer [37], glioma [38], gastric cancer [39], multiple myeloma [40] and melanoma [41]. Research also revealed that Snail1 could regulate neoangiogenesis [42].

In our research, expression level of miR-138-5p in 100 colorectal cancer tissues and cancer cell lines were examined, and in colorectal cancer, expression was decreased. Colorectal cancer tissues with and without lymph node metastasis were studied further. Results revealed that samples without lymph node metastasis were higher in expression, which was statistically significant. Results implied that miR-138-5p participated in the down regulation of the metastasis process of colorectal cancer. Scratch test showed that by administration of miR-455-3p inhibitor, colorectal cancer cell migration was promoted, which elucidated that miR-455-3p suppressed migration of colorectal cancer cell. Fluorouracial, doxorubicin, and cisplatin were used to detect chemotherapy resistance of colorectal cancer and results indicated that chemotherapy resistance could be inhibited by miR-138-5p. In addition, the difference of cell viability of LV-inhibitor + Snail1 siRNA and LV-INC + Snail1 siRNA groups was not statistically difference. NFIB was discovered as target gene of miR-138-5p using bioinformatics methods. NFIB and Snail1 were negatively correlated with miR-138-5p, while NFIB and Snail1 were positively correlated. We performed the rescue experiments to demonstrate the interactions among miR-138-5p, NFIB and Snail1. Altogether, our study unvailed that miR-138-5p targeted the NFIB-Snail1 axis to inhibit migration and chemoresistance of colorectal cancer cells.

In the present study, FHC was used as normal cell line. However, previous study indicated that FHC exhibited tumorigenic phenotype, complex karyotype, and TP53 gene mutation [43]. Therefore, other normal colon cell lines need to be applied to validate the conclusion of this study.

## Conclusions

In conclusion, we found that colorectal cancer cells were low in expression of miR-138-5p. Through targeting NFIB-Snail1 axis miR-138-5p could regulate colorectal cancer cell migration andchemoresistance. Our research shed new light in understanding colorectal cancer migration and chemoresistance. Further research is needed for miR-138-5p to be exploited for novel treatment of colorectal cancer.

## Data Availability

All data generated or analysed during this study are included in this published article.

## Abbreviations

PBS: Phosphate-buffer saline
NC: Negative control
LV-inhibitor: Lentiviral vectors containing miR-138-5p inhibitors
LV-mimic: Lentiviral vectors containing miR-138-5p mimics
