# Correction to: MicroRNA-138-5p targets the NFIB-Snail1 axis to inhibit colorectal cancer cell migration and chemoresistance

**DOI:** 10.1186/s12935-020-01629-6

**Published:** 2020-11-03

**Authors:** Weifeng Xu, Beibei Chen, Dianshan Ke, Xiaobing Chen

**Affiliations:** 1 Department of Medical Oncology, The Affiliated Cancer Hospital of Zhengzhou University, 127 Dong Ming Road, Zhengzhou, 450008 Henan People’s Republic of China; 2 Department of Cell Biology, Southern Medical University, Guangzhou, 510515 Guangdong China

## Correction to: Cancer Cell Int (2020) 20:475 https://doi.org/10.1186/s12935-020-01573-5

Following publication of the original article [1], we were notified that Dr. Xiaobing Chen should be marked as the first corresponding author.

The original article has been corrected.
